# Eight soybean reference genome resources from varying latitudes and agronomic traits

**DOI:** 10.1038/s41597-021-00947-2

**Published:** 2021-07-01

**Authors:** Jeffrey Shih-Chieh Chu, Bo Peng, Kuanqiang Tang, Xingxing Yi, Huangkai Zhou, Huan Wang, Guang Li, Jiantian Leng, Nansheng Chen, Xianzhong Feng

**Affiliations:** 1 Northeast Institute of Geography and Agroecology, Chinese Academy of Sciences, Changchun, China; 2 Wuhan Frasergen Bioinformatics Inc., East Lake High-Tech Zone, Wuhan, China; 3 Institute of Oceanology, Chinese Academy of Sciences, Qingdao, China; 4 Department of Molecular Biology and Biochemistry, Simon Fraser University, Burnaby, Canada; 5 College of Life Science and Technology, Huazhong Agricultural University, Wuhan, China

**Keywords:** Structural variation, Comparative genomics, Agricultural genetics

## Abstract

Comparative analysis of multiple reference genomes representing diverse genetic backgrounds is critical for understanding the role of key alleles important in domestication and genetic breeding of important crops such as soybean. To enrich the genetic resources for soybean, we describe the generation, technical assessment, and preliminary genomic variation analysis of eight *de novo* reference-grade soybean genome assemblies from wild and cultivated accessions. These resources represent soybeans cultured at different latitudes and exhibiting different agronomical traits. Of these eight soybeans, five are from new accessions that have not been sequenced before. We demonstrate the usage of these genomes to identify small and large genomic variations affecting known genes as well as screening for genic PAV regions for identifying candidates for further functional studies.

## Abstract (summary)

**Table:** 

Measurement(s)	DNA • RNA • sequence_assembly • genome • sequence feature annotation
Technology Type(s)	DNA sequencing • RNA sequencing • sequence assembly process • sequence annotation
Sample Characteristic - Organism	Soybean

Machine-accessible metadata file describing the reported data: 10.6084/m9.figshare.14727255

## Background & Summary

Soybean is an important crop that is responsible for about 50% of the world’s oilseed production (www.fao.org), and a source of high-quality protein for animal feeds. Cultivation of soybean has experienced specific selections for the last 4,000 years, yielding over 45,000 *Glycine max* (*G. max*) accessions^1^. The construction and subsequent targeted improvement of the reference genome assembly for *G. max* Williams 82 (W82), has greatly enhanced genome contiguity and has in turn promoted research on soybean^2,3^. However, recent genomic advances in many plant and animal species have shown that a single reference genome is insufficient to capture and represent the variations that exist within the population of the corresponding species^4–20^. Such is also true in soybeans. Resequencing and *de novo* assembly of wild and cultivated soybean accessions identified hundreds of genic presence-absence variations (PAVs) or SVs affecting agronomic genes compared to W82 genome^7,21–26^. The recent construction of the soybean pan-genome also discovered over 120 K non-redundant SVs^27^. Thus, we endeavor to enhance the genomic resources available for furthering soybean functional research and breeding by constructing reference-grade genome assemblies from cultivars found in different latitudes and exhibiting different agronomic traits. In this data descriptor, we report the sequencing, genome assembly, annotation, and genomic variation resources generated for the assembly of 8 reference-grade soybean genomes.

## Methods

### Sample selection, collection, and nucleic acid extraction

**Fig. 1:**
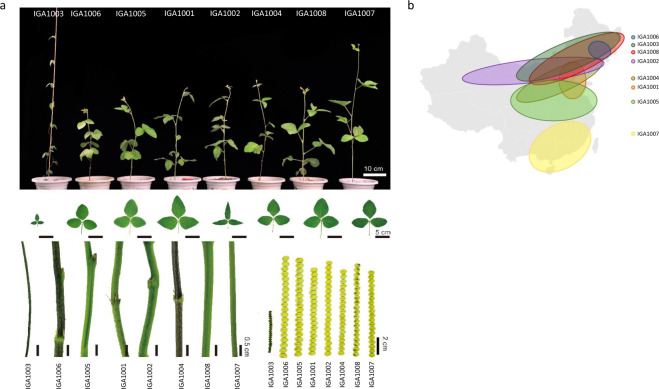
Phenotypic variation and geographical distribution of the 8 soybeans. (**a**) 8 soybean plants after growing 35 days showing differences in growth, leaf shape, stem thickness, and seed color and size; (**b**) Geographical distribution of the 8 soybeans.

Eight soybean accessions were selected for this project (Table 1). Aside from IGA1003, which is a wild soybean, the remaining seven were cultivated soybeans. These eight soybeans showed different phenotypic traits including flowering color, pubescence color, maturity time, seed shape, and hundred-grain weight (Fig. 1a, Supplementary Table 1). The eight soybeans were typically grown in different latitudes with IGA1007 and IGA1005 typically grown in southern China (around 22°N) and Huanghuai region (around 31°N), China, respectively (Fig. 1b). In terms of traits, we have included a high salinity tolerant accession (IGA1001) and a high yielding accession (IGA1004). We do note that IGA1003, IGA1005, and IGA1008 were sequenced and assembled previously. IGA1003 was sequenced and assembled using only Illumina sequencing data^7^. IGA1005^28^ and IGA1008^3^ had published genome assemblies but we have included these materials allowing for assessing genomic variations within a particular accession.

**Table 1:** Selected 8 soybean accessions.

Accession ID	Accession Name	Seed source
IGA1001	Wenfeng 7	National Soybean Preservation Center, ICS, CAAS
IGA1002	Hefeng 25	Jiamusi Branch of Heilongjiang Academy of Agricultural Sciences
IGA1003	GsojaF	National Wild Soybean Preservation Center, ICS, CAAS
IGA1004	Zhonghuang 35	National Soybean Improvement Sub-Center, ICS, CAAS
IGA1005	Zhonghuang 13	National Soybean Improvement Sub-Center, ICS, CAAS
IGA1006	Jingyuan	National Soybean Preservation Center, ICS, CAAS
IGA1007	Huaxia 3	South China Agricultural University
IGA1008	Williams 82	Northeast Institute of Geography and Agroecology, CAS

Abbreviations: ICS: Institute of Crop Sciences; CAAS: Chinese Academy of Agricultural Sciences; CAS: Chinese Academy of Sciences.

Seeds from each accession were grown in the greenhouse at Northeast Institute of Geography and Agroecology. Five seeds were planted with 14 hours of light and temperature controlled at 28 °C during the day time and 20 °C during the night time. Soil composition was 8:5:3 ratio of peat:vermiculite:perlite mixed with 5 g of phosphate. Plants were grown until the V2 growth phase and the leaves were harvested from the top.

Genomic DNA was extracted from 1 g of leaf tissue harvested from V2 growth period using CTAB method. Quality of the genomic DNA was assessed using the Qubit Fluorometric system (ThermoFisher) and gel electrophoresis system. Total RNA was isolated from root, stem and leaf separately using TRIzol. Quality of the RNA was assessed using Qubit Fluorometric system and Agilent 2100 Bioanalyzer (Agilent). A quality of RIN >7 was considered high quality RNA.

### DNA sequencing library construction and sequencing

DNA fragments >20 kb was selected for PacBio library preparation using BluePippin (SAGE). PacBio library was prepared using SMRTbell Template Prep Kit-SPv3 following manufacturer’s recommendations (Pacific Biosciences). The library QC was performed using Qubit and Agilent 2100. The final library was sequenced on the Pacific Biosciences Sequel system. For Illumina sequencing library construction, the genomic DNA was fragmented to 300–500 bp using Covaris M220 (Covaris). Illumina library was prepared using NEBNext Ultra II DNA Library Prep Kit for Illumina following manufacturer’s recommendations (NEB) and sequenced on the Illumina system with PE150 format. We generated 4–6.74 million PacBio reads^29–36^ with average subread length N50 of 14Kb and 296–368 million Illumina short reads (Table 2)^37–44^. The estimated sequencing depth based on soybean genome size was 50X Illumina short reads and 50X of PacBio long reads.

**Table 2:** Sequencing output of each library type for the 8 soybean accessions.

Accession ID	PacBio raw data (G)	PacBio number of subreads (M)	Subreads length N50 (Kb)	Illumina DNA raw data (G)	Illumina RNA raw data (G)	Hi-C library raw data (G)
IGA1001	48.51	6.74	12.52	43.2	11.37	49.80
IGA1002	51.14	6.37	12.68	53.6	10.44	57.81
IGA1003	51.97	6.54	12.27	52.3	11.54	46.43
IGA1004	48.01	5.50	12.53	47.8	13.72	54.67
IGA1005	50.10	4.50	16.56	57.6	14.62	57.14
IGA1006	58.04	6.13	13.84	48.9	12.91	51.05
IGA1007	49.69	4.01	18.56	51.6	11.88	52.82
IGA1008	53.64	6.74	12.47	46.8	12.77	54.45

### RNA sequencing library construction and sequencing

RNA from each tissue was pooled in equal molar to produce a mixed RNA sample. mRNA was enriched using Poly(A) mRNA Magnetic Isolation kit (NEB) followed by RNA-seq library construction using NEBNext Ultra II RNA Library Prep Kit for Illumina (NEB) following manufacturer’s recommendations. Sequencing was performed on the Illumina system with PE150 format to generate 10.43–14.61 Gb of data (Table 2)^45–52^.

### Hi-C sample preparation, library construction and sequencing

About 1.5 g of young leaves were used for Hi-C library construction as described in previous reports with some modifications^53^. Briefly, leaf samples were cross-linked with 3% formaldehyde for 45 minutes in vacuum at 4 °C and stopped using 0.4 M glycine. Leaf pellets were then pulverized in liquid nitrogen followed by resuspension in the nuclei isolation buffer (NIB). The cross-linked nuclei were treated with 0.3% SDS and neutralized with 3% Triton X-100. The resulting DNA was digested with MboI (NEB) overnight at 37 °C and the reaction was stopped with heat inactivation at 65 °C. Restriction fragment ends were fixed with Klenow and labeled with biotinylated cytosine nucleotides using biotin-14-dCTP (TriLINK). Blunt-end ligation was carried out using T4 DNA ligase incubated at 16 °C overnight. After ligation, the cross-linking was reversed by proteinase K (Thermo) overnight at 65 °C. DNA purification was performed using DNeasy Plant Mini Kit (Qiagen) according to manufacturer’s instructions. Purified DNA was sheared to a length of ~400 bp using Covaris M220 (Covaris). Hi-C ligated junctions were captured by Dynabeads MyOne Streptavidin C1 (Thermofisher) according to manufacturer’s instructions. The Hi-C sequencing library was prepared using NEBNext Ultra II DNA library Prep Kit for Illumina (NEB) following manufacturer’s instructions. Fragments between 400 and 600 bp were sequenced on the Illumina platform with PE150 format to generate 46–57 G of data (Table 2)^54–61^.

### *De novo* genome assembly

*De novo* assembly was performed with PacBio sequencing data using FALCON^62^ setting length_cutoff = 3000–13000 and length_cutoff_pr = 3000 and CANU^63^ setting correctedErrorRate = 0.039–0.04. We further improved the assembly by merging complementing contigs between FALCON and CANU using CANU assembly as the basis^64^. The final contigs were assembled into chromosomes with Hi-C data using LACHESIS v.c23474f^65^. The assembly was corrected with PacBio long reads using Arrow in SMRTLink 5.0^66^ and Illumina short reads using Pilon v1.22^67^. The final genome assemblies ranged between 986.1 Mb and 1001.3 Mb with more than 98.7% of the genome anchored to 20 chromosomes. The contig N50 were between 1.4 Mb–6.1 Mb and the scaffold N50 were between 48.8 Mb–50.7 Mb (Table 3)^68–75^.

**Table 3:** Genome assembly, BUSCO evaluation and remapping assessment of the 8 soybean accessions.

	Assembly Length (Mb)	Number of contigs	Contig N50 (Mb)	Number of Scaffolds	Scaffold N50 (Mb)	Number of Gaps	BUSCO %	Het SNPs (%)	Hom SNPs (%)	NCBI Accession
IGA1001	996	2623	1.74	334	50.7	2289	96.9	0.0055	0.0005	WIWY00000000
IGA1002	987	1968	2.92	249	49.51	1719	97	0.0051	0.0003	WIWX00000000
IGA1003	975	2406	1.55	320	48.77	2086	97.2	0.0088	0.0005	WIXD00000000
IGA1004	1001	3382	1.41	460	50.49	2922	97	0.0213	0.0005	WIWZ00000000
IGA1005	988	1169	4.65	181	50.28	988	97.1	0.0037	0.0002	WIXA00000000
IGA1006	995	1420	4.27	332	50.57	1088	96.7	0.004	0.0003	WIXB00000000
IGA1007	986	1038	6.16	208	50.07	830	96.7	0.0039	0.0002	WIXC00000000
IGA1008	993	2339	1.88	397	49.8	1942	97.3	0.0061	0.0005	WIWW00000000

Abbreviations: Het: Heterozygous; Hom: Homozygous.

### Genome annotation

Repetitive elements were first annotated before other features. Tandem Repeat Finder v.4.09^76^ was used to annotate tandem repeats. LTR_FINDER^77^ was used to build a LTR-retrotransponson library and RepeatModeler v.1.0.10 (http://www.repeatmasker.org/RepeatModeler.html) was used to build a de novo repetitive element library. The above libraries and Repbase^78^ were used by RepeatMasker^79^ to annotate repetitive elements. About 50% of each genome was annotated with repetitive elements (Table 4), which is similar to published soybean genomes^3,22,28^. After repetitive sequences were masked, protein-coding gene annotation was performed utilizing *ab initio*-, homology-, RNA-sequencing-, and Iso-seq-based methods. Augustus v.3.3^80^ and Glimmer v.3.0.4^81^ were used for *ab initio* gene prediction. For homology-based annotation, protein sequences from *Glycine max* Williams 82, *Glycine soja*, *Arabidopsis thaliana*, *Arachis duranensis*, and *Cajanus cajan* were obtained from NCBI and aligned to each of the 8 genomes using TBLASTN^82^. Exonerate was used to build gene structure based on the Blast results. For RNA-seq based gene prediction, short reads were mapped to their respective genome using TopHat v2.1.1^83^ and the gene structure was predicted using Cufflinks v2.2.1^84^. For Iso-seq based gene prediction, reads were mapped to IGA1008 assembly using GMAP v2016-09-14^85^ and TransDecoder v4.1.0 was used to filter for high quality gene models. Lastly, a consensus gene set was generated by integrating gene annotations from each source using MAKER^86^. With this process, we identified 57,286 to 58,392 protein coding genes (Table 4).

**Table 4:** Genome annotation and BUSCO assessment of the 8 soybean accessions.

Accession		IGA1001	IGA1002	IGA1003	IGA1004	IGA1005	IGA1006	IGA1007	IGA1008
**Protein coding genes**		57505	58102	57961	58150	57474	58392	57396	57286
**Complete BUSCO %**		95.3	95.1	96.9	95.4	95.2	93.4	95.5	95.9
**Non-coding genes**	miRNA	279	282	283	286	277	281	279	280
	tRNA	1028	1163	1122	1028	1077	1100	1060	999
	rRNA	246	383	233	139	336	315	136	202
	snRNA	2645	2609	2643	2709	2612	2609	2605	2617
**Repetitive elements**	LTR-Retro-transposons (%)	39.74	39.2	38.78	39.39	39.84	40.05	39.55	39.6
	LINE (%)	1.93	1.95	1.87	1.84	1.87	1.8	1.81	1.91
	SINE (%)	0.07	0.05	0.08	0.1	0.06	0.04	0.04	0.07
	DNA	7.11	7.24	7.01	7.37	7.03	7.02	7.17	7.3
	Transposons (%)								
	Satellites (%)	0.12	0.11	0.35	0.1	0.11	0.32	0.11	0.43
	Simple repeats (%)	1.23	1.2	0.67	0.98	1.03	1.51	0.92	1.01
	Total (%)	50.2	49.75	48.76	49.78	49.94	50.74	49.6	50.32

Non-coding RNA (ncRNA) annotation was performed using INFERNAL^87^ based on Rfam v.14.1 models. tRNA was annotated using tRNAscan-SE v.1.3.1^88^. rRNA annotation was performed using BLASTN with known soybean rRNA sequences.

### BUSCO evaluation of genome and annotations

BUSCO (Benchmarking Universal Single-Copy Orthologs) evaluation was performed on the genome assembly and the gene annotations using BUSCO v.3.0.2^89^ with embryophyta_odb9 data set.

### Variation detection

Whole genome alignment based variation detection was performed by aligning each genome to IGA1008 using MUMer4^90^ with parameters–mum –l 40 –c 90. The MUMer alignment was filtered using delta-filter with parameters --1. SNVs, small Indels were called using show-snps with parameters -rlTHC. Large SVs were called using custom scripts MumSV (https://github.com/jeff-sc-chu/MumSV) based on output from show-coords with parameters -THrcl. Alignment gaps associated with assembly gaps were filtered away. Presence-absence variations (PAVs) were assessed based on large insertions and deletions (>100 bp) using BLASTN against the reference genome. A deletion or insertion is considered a PAV if BLASTN alignment to the reference genome is <50% query coverage or <90% PID. Accession specific SNP, Indel, and SVs were identified by selecting variants not overlapping any variants of the same class in all other genomes. Using the above process, we identified 1.86–4.27 million SNVs, 0.44–0.92 million small indels, 11.75–25.33 thousand large indels (>100 bp), 706–3006 translocation events, and 200–413 inversion events (Table 5).

**Table 5:** Genomic variation of 7 soybean accessions compared with IGA1008.

Accession	IGA1001	IGA1002	IGA1003	IGA1004	IGA1005	IGA1006	IGA1007
**SNPs**							
Total SNPs	2429031	1860802	4275544	2321059	1938310	1970083	2121229
**Indels**							
Total Indels	563950	462627	922735	565841	478827	441705	495694
**Structural Variations**							
Large SV indels	14747	11750	25335	14083	11933	12340	12980
Translocations	1213	726	3006	1142	706	950	885
Inversions	290	223	413	393	200	210	208
**PAVs**							
PV events	2004	1593	3575	1869	1659	1504	1780
AV events	1905	1515	3330	1851	1586	1469	1692

## Data Records

The sequencing data, genome assembly, and genome annotation of the 8 soybeans have been deposited in NCBI under the BioProject PRJNA561626. This includes whole genome sequencing data from the PacBio Sequel platform^29–36^ and from the Illunina platform^37–44^; RNA sequencing data from the Illumina platform^45–52^; Hi-C library sequencing data from the Illumina platform^54–61^; and genome assembly sequences and annotations^68–75^. The individual BioProject, BioSample, and SRA accession IDs are also listed in Supplementary Table 2. Gene annotations in GFF3 format, one-to-one gene correspondences between IGA1008 and W82_v4, and the coordinates of SNPs, Indels, and PAVs are available on Figshare with 10.6084/m9.figshare.c.5106161.v4^91^. We have also detailed the resources available on Figshare in Table 6. The plant materials used in this study are available from the authors upon request.

**Table 6:** Description of file hosted on the Figshare record (10.6084/m9.figshare.c.5106161.v4) accompanying this paper.

Title	Description
Annotated peptide sequence for each soybean genome	Peptide sequence of each annotated coding gene for each genome
Annotated CDS sequences for each soybean genome	Coding sequences of each annotated gene for each genome
Gene annotations (GFF)	Gene annotation of each genome in GFF format
SNVs for each genome compared to IGA1008	SNV location detected by whole genome MUMer alignment of each genome using IGA1008 as a reference.
Indels between soybean genome assemblies and IGA1008	Indel location detected by whole genome MUMer alignment of each genome using IGA1008 as a reference.
PAVs of between soybean genomes compared with IGA1008	PAV location detected by whole genome MUMer alignment of each genome using IGA1008 as a reference.
Gene orthology correspondence	Gene orthology correspondence between IGA1008 and Williams 82 v4 genome.

## Technical Validation

### Genome assembly quality assessment

The final assemblies of the 8 genomes ranged between 986.1 Mb and 1001.3 Mb with more than 98.7% of the genome anchored to 20 chromosomes (Table 3). The genome assembly size is similar to its respective k-mer estimation (Table 3) and also comparable to soybean genomes, such as W82_v4^3^, ZH13^28^, and W05^26^, which were assembled using similar sequencing technologies. The contig N50 were between 1.4 Mb–6.1 Mb and the scaffold N50 were between 48.8 Mb–50.7 Mb (Table 3)^68–75^. The number of gaps ranged between 830 and 2922 (Table 3). Each unspanned gap in the assembly was arbitrarily set with 500 bp of Ns. As a comparison, the current soybean reference Williams 82 version 4 genome has a genome size of 978 Mb with contig N50 of 0.41 Mb, scaffold N50 of 20.44 Mb, and 8920 gaps^3^.

We further assessed the quality of our genome assemblies in a number of ways. First, the presence of centrimeric repeats CentGm-1/2 were found in all 20 chromosomes for all genome assemblies. We assessed the completion and accuracy of the assemblies using BUSCO^89^ and re-mapping of Illumina short read data. We observed an average of 96.98% complete BUSCO alignment and an average 99.75% remapping rate. Next, a high accuracy genome assembly would expect a very low level of homozygous SNVs from the remapping analysis and as expected we see an average of 0.00037% homozygous SNVs and 0.0073% heterozygous SNVs indicating the low error rates in these assemblies (Table 3).

### Genome comparison with soybean reference genome Williams 82

IGA1008 is a soybean cultivar derived from Williams 82. Between IGA1008 and the W82_v4 assemblies, we identified 0.38 million SNVs, 0.14 million small indels, 3,203 large indels (>100 bp), 255 translocation-like and 135 inversion-like events. These events do not border or cross assembly gaps and may represent genomic variations between the two W82 lines.

### Gene set completeness assessment

The numbers of protein-coding genes annotated for these cultivars were highly similar, ranging between 57,286 and 58,392 genes. We used BUSCO to evaluate the completion of our gene annotation and found that over 96% of the 1440 genes were found completely (Table 4).

### Structural variation assessment

IGV^92^ was used to visually inspect many structural variations. We also examined known structural variations for their presence in the 8 genomes and our data confirmed the 40Kb Williams 82-specific insertion on Chromosome 15 (Supplementary Fig. 1)^7,26^, the I-locus inversion event (Supplementary Fig. 2)^26^ and the large deletion (~15Kb) affecting the E3 gene (Supplementary Fig. 3)^27^.

## Usage Notes

### Identifying variations in known genes

The resources generated in this dataset allows one to search for genomic variations in genes of interest. For instance, we were able to identify nonsense mutations in the E2 gene as well as frameshift indels in J and FT1b (Supplementary Fig. 3). These resources, such as the indel found in FT1b, has not been characterized before and provide candidate alleles for further soybean research.

### PAV gene screening

**Fig. 2:**
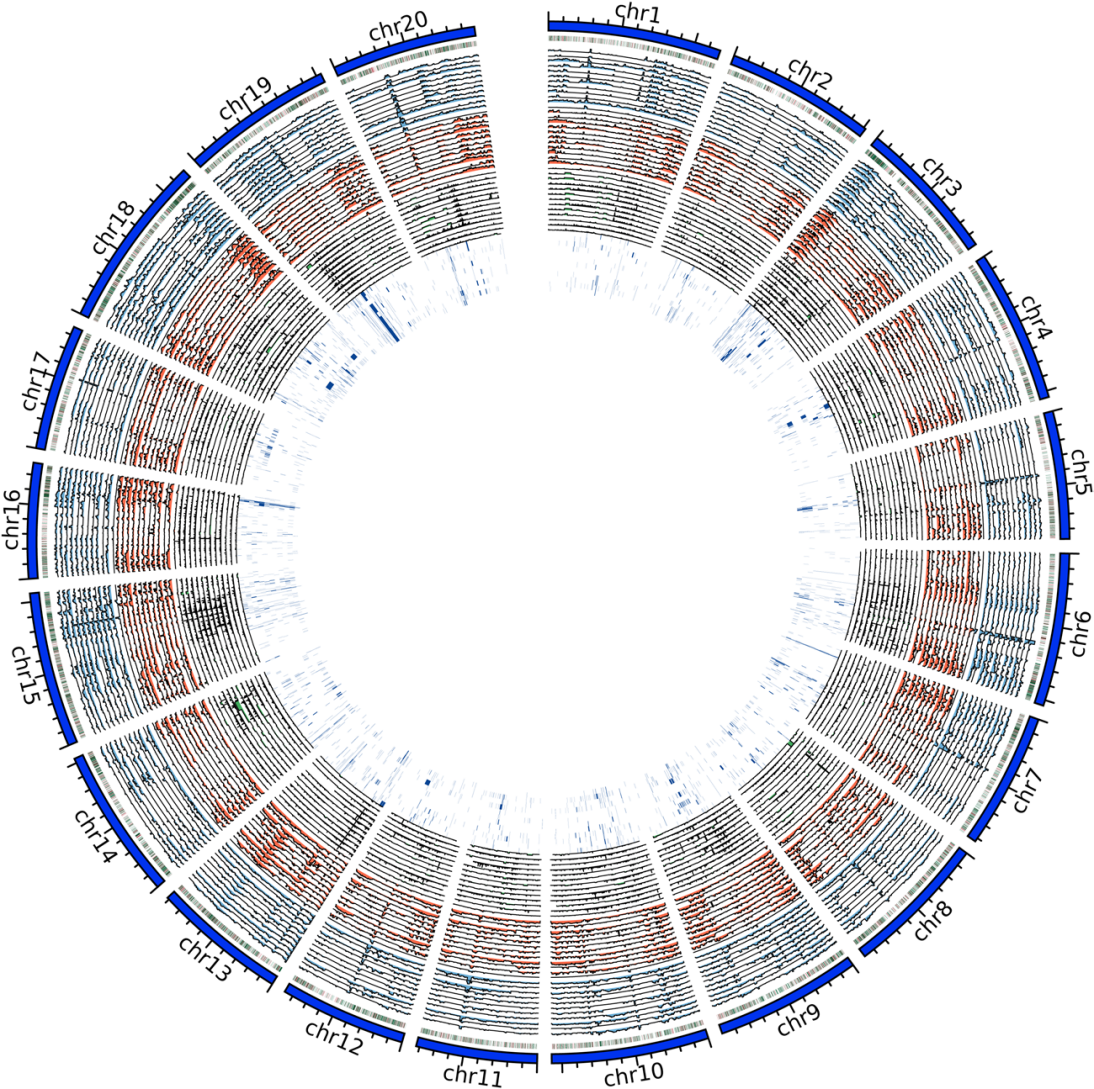
Genomic variations of 12 soybeans compared to IGA1008 genome. The rings are in the following order from outer-most to inner-most: IGA1008 chromosomes (navy blue), PAV regions with dark red representing PV and dark green representing AV, a set of blue histograms represent SNP density in 400Kb window, the set of orange histograms represent small indel density in 400Kb window, the set of green histograms represent the large indel sizes, the inner-most set of dark blue highlights represent inversions. Each set depicts the variation of 12 soybeans ordered from outside to inside: IGA1001, IGA1002, IGA1003, IGA1004, IGA1005, IGA1006, IGA1007, ZH13, W05, Lee, PI483463, W82_v4.

As more soybean genomic resources become available, one can take a pan-genome approach to identify genomic variations that are unique or shared between different soybean accessions. As a demonstration, we included 5 additional soybean genomes (ZH13^28^, W05^26^, Lee^3^, PI483463^3^, W82^3^) to identify genomic variations that are shared in a subset of the genomes (Fig. 2). These 5 genomes were chosen for their genome assembly quality, which were improved using sequencing technologies with longer reads. We identified 60 genes that were found in 3 wild soybeans but missing or truncated in the other 10 cultivated soybeans and 185 genes found missing or truncated in all 3 wild soybeans (Supplementary Table 3). Analyses such as this could generate additional resources to further soybean research and crop improvement.

## Supplementary information

Supplementary Information

## Data Availability

The versions and parameters of published software used in this study were described in the Methods. MumSV is a set of custom scripts to call large SVs based on MUMer alignments and it can be accessed at https://github.com/jeff-sc-chu/MumSV.

## References

[1] Dashiell, K. SOYBEANS: Improvement, Production, and Uses. Third Edition. *Agricultural Systems* vol. 83 110–111 (2005).

[2] Schmutz J (2010). Genome sequence of the palaeopolyploid soybean. Nature.

[3] Valliyodan B (2019). Construction and comparison of three reference-quality genome assemblies for soybean. Plant J..

[4] Golicz AA (2016). The pangenome of an agronomically important crop plant Brassica oleracea. Nat. Commun..

[5] Da Silva C (2013). The high polyphenol content of grapevine cultivar tannat berries is conferred primarily by genes that are not shared with the reference genome. Plant Cell.

[6] Li M (2017). Comprehensive variation discovery and recovery of missing sequence in the pig genome using multiple de novo assemblies. Genome Res..

[7] Li Y-H (2014). De novo assembly of soybean wild relatives for pan-genome analysis of diversity and agronomic traits. Nat. Biotechnol..

[8] Malinsky M (2018). Whole-genome sequences of Malawi cichlids reveal multiple radiations interconnected by gene flow. Nat Ecol Evol.

[9] Sherman RM (2019). Assembly of a pan-genome from deep sequencing of 910 humans of African descent. Nat. Genet..

[10] Gan X (2011). Multiple reference genomes and transcriptomes for Arabidopsis thaliana. Nature.

[11] Read BA (2013). Pan genome of the phytoplankton Emiliania underpins its global distribution. Nature.

[12] Yu J (2019). Insight into the evolution and functional characteristics of the pan-genome assembly from sesame landraces and modern cultivars. Plant Biotechnol. J..

[13] Zhang Q-J (2014). Rapid diversification of five Oryza AA genomes associated with rice adaptation. Proc. Natl. Acad. Sci. USA.

[14] Neafsey DE (2015). Mosquito genomics. Highly evolvable malaria vectors: the genomes of 16 Anopheles mosquitoes. Science.

[15] Hu Z (2018). Novel sequences, structural variations and gene presence variations of Asian cultivated rice. Sci Data.

[16] Wang W (2018). Genomic variation in 3,010 diverse accessions of Asian cultivated rice. Nature.

[17] Stein JC (2018). Genomes of 13 domesticated and wild rice relatives highlight genetic conservation, turnover and innovation across the genus Oryza. Nat. Genet..

[18] Yao W (2015). Exploring the rice dispensable genome using a metagenome-like assembly strategy. Genome Biol..

[19] Kehr B (2017). Diversity in non-repetitive human sequences not found in the reference genome. Nat. Genet..

[20] Song J-M (2020). Eight high-quality genomes reveal pan-genome architecture and ecotype differentiation of Brassica napus. Nat Plants.

[21] Valliyodan B (2016). Landscape of genomic diversity and trait discovery in soybean. Sci. Rep..

[22] Shen Y (2018). De novo assembly of a Chinese soybean genome. Sci. China Life Sci..

[23] Shimomura M (2015). The Glycine max cv. Enrei Genome for Improvement of Japanese Soybean Cultivars. Int. J. Genomics Proteomics.

[24] Kim MY (2010). Whole-genome sequencing and intensive analysis of the undomesticated soybean (Glycine soja Sieb. and Zucc.). genome. Proc. Natl. Acad. Sci. USA.

[25] Qi X (2014). Identification of a novel salt tolerance gene in wild soybean by whole-genome sequencing. Nat. Commun..

[26] Xie M (2019). A reference-grade wild soybean genome. Nat. Commun..

[27] Liu Y (2020). Pan-Genome of Wild and Cultivated Soybeans. Cell.

[28] Shen Y (2019). Update soybean Zhonghuang 13 genome to a golden reference. Sci. China Life Sci..

[29] (2020). NCBI Sequence Read Archive.

[30] (2020). NCBI Sequence Read Archive.

[31] (2020). NCBI Sequence Read Archive.

[32] (2020). NCBI Sequence Read Archive.

[33] (2020). NCBI Sequence Read Archive.

[34] (2020). NCBI Sequence Read Archive.

[35] (2020). NCBI Sequence Read Archive.

[36] (2020). NCBI Sequence Read Archive.

[37] (2020). NCBI Sequence Read Archive.

[38] (2020). NCBI Sequence Read Archive.

[39] (2020). NCBI Sequence Read Archive.

[40] (2020). NCBI Sequence Read Archive.

[41] (2020). NCBI Sequence Read Archive.

[42] (2020). NCBI Sequence Read Archive.

[43] (2020). NCBI Sequence Read Archive.

[44] (2020). NCBI Sequence Read Archive.

[45] (2020). NCBI Sequence Read Archive.

[46] (2020). NCBI Sequence Read Archive.

[47] (2020). NCBI Sequence Read Archive.

[48] (2020). NCBI Sequence Read Archive.

[49] (2020). NCBI Sequence Read Archive.

[50] (2020). NCBI Sequence Read Archive.

[51] (2020). NCBI Sequence Read Archive.

[52] (2020). NCBI Sequence Read Archive.

[53] Rao SSP (2014). A 3D map of the human genome at kilobase resolution reveals principles of chromatin looping. Cell.

[54] (2020). NCBI Sequence Read Archive.

[55] (2020). NCBI Sequence Read Archive.

[56] (2020). NCBI Sequence Read Archive.

[57] (2020). NCBI Sequence Read Archive.

[58] (2020). NCBI Sequence Read Archive.

[59] (2020). NCBI Sequence Read Archive.

[60] (2020). NCBI Sequence Read Archive.

[61] (2020). NCBI Sequence Read Archive.

[62] Chin C-S (2016). Phased diploid genome assembly with single-molecule real-time sequencing. Nat. Methods.

[63] Koren S (2017). Canu: scalable and accurate long-read assembly via adaptive -mer weighting and repeat separation. Genome Res..

[64] Zhang J (2016). Genome puzzle master (GPM): an integrated pipeline for building and editing pseudomolecules from fragmented sequences. Bioinformatics.

[65] Burton JN (2013). Chromosome-scale scaffolding of de novo genome assemblies based on chromatin interactions. Nat. Biotechnol..

[66] Chin C-S (2013). Nonhybrid, finished microbial genome assemblies from long-read SMRT sequencing data. Nat. Methods.

[67] Walker BJ (2014). Pilon: an integrated tool for comprehensive microbial variant detection and genome assembly improvement. PLoS One.

[68] Feng X (2021). GenBank.

[69] Feng X (2021). GenBank.

[70] Feng X (2021). GenBank.

[71] Feng X (2021). GenBank.

[72] Feng X (2021). GenBank.

[73] Feng X (2021). GenBank.

[74] Feng X (2021). GenBank.

[75] Feng X (2021). GenBank.

[76] Benson G (1999). Tandem repeats finder: a program to analyze DNA sequences. Nucleic Acids Research.

[77] Xu Z, Wang H (2007). LTR_FINDER: an efficient tool for the prediction of full-length LTR retrotransposons. Nucleic Acids Res..

[78] Bao W, Kojima KK, Kohany O (2015). Repbase Update, a database of repetitive elements in eukaryotic genomes. Mob. DNA.

[79] Tarailo-Graovac M, Chen N (2009). Using RepeatMasker to identify repetitive elements in genomic sequences. Curr. Protoc. Bioinformatics.

[80] Stanke M (2006). AUGUSTUS: ab initio prediction of alternative transcripts. Nucleic Acids Res..

[81] Delcher AL, Bratke KA, Powers EC, Salzberg SL (2007). Identifying bacterial genes and endosymbiont DNA with Glimmer. Bioinformatics.

[82] Altschul SB (1990). Local Alignment Search Tool. Journal of Molecular Biology.

[83] Trapnell C, Pachter L, Salzberg SL (2009). TopHat: discovering splice junctions with RNA-Seq. Bioinformatics.

[84] Trapnell C (2010). Transcript assembly and quantification by RNA-Seq reveals unannotated transcripts and isoform switching during cell differentiation. Nat. Biotechnol..

[85] Wu TD, Watanabe CK (2005). GMAP: a genomic mapping and alignment program for mRNA and EST sequences. Bioinformatics.

[86] Campbell MS (2014). MAKER-P: a tool kit for the rapid creation, management, and quality control of plant genome annotations. Plant Physiol..

[87] Nawrocki EP, Eddy SR (2013). Infernal 1.1: 100-fold faster RNA homology searches. Bioinformatics.

[88] Chan PP, Lowe TM (1962). tRNAscan-SE: Searching for tRNA Genes in Genomic Sequences. Methods Mol. Biol..

[89] Seppey M, Manni M, Zdobnov EM (2019). BUSCO: Assessing Genome Assembly and Annotation Completeness. Methods Mol. Biol..

[90] Kurtz S (2004). Versatile and open software for comparing large genomes. Genome Biol..

[91] Chu J (2021). figshare.

[92] Robinson JT, Thorvaldsdóttir H, Wenger AM, Zehir A, Mesirov JP (2017). Variant Review with the Integrative Genomics Viewer. Cancer Res..

