# Supplementary material for: Eight soybean reference genome resources from varying latitudes and agronomic traits
Source: Sci Data. 2021 Jul 1;8:164. doi: 10.1038/s41597-021-00947-2 (PMC8249447; doi:10.1038/s41597-021-00947-2)
Supplement: Supplementary file 1 — Supplementary Information [file 41597_2021_947_MOESM1_ESM.pdf]

Supplementary Information
for
Eight soybean reference genomes resources from varying latitudes and agronomic traits

Jeffrey Shih-Chieh Chu[1,2], Bo Peng[2], Kuanqiang Tang[1], Xingxing Yi[2,5], Huangkai Zhou[1], Huan Wang[2], Guang Li[1], Jiantian Leng[1], Nansheng Chen[3,4*], Xianzhong Feng[1*]

Table of Contents

Supplementary Table 1. Agronomic traits of the eight soybean accessions. 2

Supplementary Table 2. SRA accessions of PacBio, Illumina DNA, and RNA sequencing for each sample. 3

Supplementary Table 3. List of genes found to be either wild-specific or wild-missing.................... 4

Supplementary Figure 1. Williams 82 specific PAV on chr15 compared with each genome..................... 10

Supplementary Figure 2. Inversion variation on I locus in new genomes compared with IGA1008 11

Supplementary Figure 3. Genomic variations found in a) E3 gene, b) E2 gene, c) J gene, d) FT1b gene 12

Supplementary Table 1. Agronomic traits of the eight soybean accessions.

**Accession ID name**	**IGA1001**	**IGA1002**	**IGA1003**	**IGA1004**	**IGA1005**	**IGA1006**	**IGA1007**	**IGA1008**
**Flowering time (days)**	48	30	28	56	50	45	73	68
**Growth Period (days)**	120	118	70	121	97	115	113	128
**Leaf Shape**	Round	Pointed	Oval	Round	Oval	Round	Round	Oval
**Flower color**	White	White	Purple	White	Purple	White	White	White
**pubescence color**	Gray	Gray	Brown	Gray	Gray	Gray	Brown	Brown
**Pod habit**	limited	limited	unlimited	limited	limited	limited	limited	unlimited
**Plant shape**	Erect	Erect	Viney	Erect	Erect	Erect	Erect	Erect
**Pod shattering**	resistant	resistant	Shatters	resistant	resistant	resistant	resistant	resistant
**Lodging**	0	0		0	0	0	level 1	0
**Disease susceptibility**	none	none	none	none	none	none	none	none
**Plant height (cm)**	71.3	75.3	60	67.3	46.3	80.3	84.7	95.8
**Bottom pod height**	15	10	5	28	25	15	20	18
**Nodes**	14	13		15	13.8	14	19	18
**Branches**	3.2	0	9.8	1.8	4.3	2.1	4.5	5.6
**node position**	5\6\7\8\9	\	>4 nodes	5\6\7	5\6\7\8\9\10	5\6\7\8	7\8\9\10\11\12	5\6\7\8\9\10\11
**Pods per plant**	35	38	45	50.6	40	38	145.7	89
**Seeds per plant**	83	98	68	113	79	91	149	178
**Seeds per pod**	2.5	3.1	2.6	2.4	2.04	2.8	2.6	2.5
**Seed shape**	Oval	Oval	Near Round	Round	Oval	Round	Oval	Oval
**Seed color**	Yellow	Yellow	Black	Yellow	Yellow	Yellow	Yellow	Light Yellow
**hilum color**	Brown	Light color	Black	Yellow	Light brown	Light	Light brown	Black
**Hundred seed weight (g)**	16.3	21.3	1.15	18.5	22	20.4	17.5	19.9

Supplementary Table 2. SRA accessions of PacBio, Illumina DNA, and RNA sequencing for each sample.

Soybean Accession ID	LibraryName	BioProject	BioSample	Sequencing data accession
IGA1002	G. max Hefeng 25_Hi-C	PRJNA561626	SAMN12622429	SRX7010335
IGA1002	G. max Hefeng 25 _ILLUMINA	PRJNA561626	SAMN12622413	SRX6983681
IGA1002	G. max Hefeng 25_PacBio	PRJNA561626	SAMN12622413	SRX7016449
IGA1002	G. max Hefeng 25_RNAseq	PRJNA561626	SAMN12622421	SRX7010783
IGA1007	G. max Huaxia 3_Hi-C	PRJNA561626	SAMN12622434	SRX7010338
IGA1007	G. max Huaxia3_PacBio	PRJNA561626	SAMN12622418	SRX7010599
IGA1007	G. max Huaxia 3 _ILLUMINA	PRJNA561626	SAMN12622418	SRX7009429
IGA1007	G. max Huaxia 3_RNAseq	PRJNA561626	SAMN12622426	SRX7010779
IGA1006	G. max Jinyuan_Hi-C	PRJNA561626	SAMN12622433	SRX7010337
IGA1006	G. max Jinyuan _ILLUMINA	PRJNA561626	SAMN12622417	SRX7009431
IGA1006	G. max Jinyuan_PacBio	PRJNA561626	SAMN12622417	SRX7010600
IGA1006	G. max Jinyuan_RNAseq	PRJNA561626	SAMN12622425	SRX7010782
IGA1001	G. max Wenfeng 7_Hi-C	PRJNA561626	SAMN12622430	SRX7026256
IGA1001	G. max Wenfeng 7_PacBio	PRJNA561626	SAMN12622414	SRX7026392
IGA1001	G. max Wenfeng 7_ILLUMINA	PRJNA561626	SAMN12622414	SRX6825670
IGA1001	G. max Wenfeng7_RNAseq	PRJNA561626	SAMN12622422	SRX7016199
IGA1008	G. max Williams 82_Hi-C	PRJNA561626	SAMN12622428	SRX7010339
IGA1008	G. max Williams 82 _ILLUMINA	PRJNA561626	SAMN12622412	SRX7009428
IGA1008	G. max Williams 82_PacBio	PRJNA561626	SAMN12622412	SRX7016855
IGA1008	Glycine max_isoseq	PRJNA561626	SAMN12622420	SRX7016448
IGA1008	G. max Williams 82_RNAseq	PRJNA561626	SAMN12622420	SRX7010787
IGA1005	G. max Zhonghuang 13_Hi-C	PRJNA561626	SAMN12622432	SRX7010348
IGA1005	G. max Zhonghuang 13_ILLUMINA	PRJNA561626	SAMN12622416	SRX7009433
IGA1005	G. max Zhonghuang 13_PacBio	PRJNA561626	SAMN12622416	SRX7010602
IGA1005	G. max Zhonghuang 13_RNAseq	PRJNA561626	SAMN12622424	SRX7010786
IGA1004	G. max Zhonghuang 35_Hi-C	PRJNA561626	SAMN12622431	SRX7010349
IGA1004	G. max Zhonghuang 35_PacBio	PRJNA561626	SAMN12622415	SRX7010601
IGA1004	G. max Zhonghuang 35_ILLUMINA	PRJNA561626	SAMN12622415	SRX7009430
IGA1004	G. max Zhonghuang 35_RNAseq	PRJNA561626	SAMN12622423	SRX7010784
IGA1003	G. soja F_Hi-C	PRJNA561626	SAMN12622435	SRX7010336
IGA1003	G. max G. soja F_ILLUMINA	PRJNA561626	SAMN12622419	SRX7009432
IGA1003	G. soja F_PacBio	PRJNA561626	SAMN12622419	SRX7010603
IGA1003	G. soja F_RNAseq	PRJNA561626	SAMN12622427	SRX7010785

Supplementary Table 3. List of genes found to be either wild-specific or wild-missing.

Gene ID	Gene status in the 3 wild soybeans
SoyGsojaF_11R054271	Wild-specific
SoyGsojaF_11R054565	Wild-specific
SoyGsojaF_12R051260	Wild-specific
SoyGsojaF_12R051259	Wild-specific
SoyGsojaF_12R051261	Wild-specific
SoyGsojaF_12R051375	Wild-specific
SoyGsojaF_12R051504	Wild-specific
SoyGsojaF_12R051503	Wild-specific
SoyGsojaF_12R051501	Wild-specific
SoyGsojaF_12R051499	Wild-specific
SoyGsojaF_12R051502	Wild-specific
SoyGsojaF_12R051505	Wild-specific
SoyGsojaF_12R051500	Wild-specific
SoyGsojaF_12R052051	Wild-specific
SoyGsojaF_12G092800	Wild-specific
SoyGsojaF_12R051085	Wild-specific
SoyGsojaF_14R021516	Wild-specific
SoyGsojaF_14R023057	Wild-specific
SoyGsojaF_14R023147	Wild-specific
SoyGsojaF_14R023148	Wild-specific
SoyGsojaF_14R023158	Wild-specific
SoyGsojaF_14R023159	Wild-specific
SoyGsojaF_14R023157	Wild-specific
SoyGsojaF_14R023233	Wild-specific
SoyGsojaF_14G187300	Wild-specific
SoyGsojaF_14G199500	Wild-specific
SoyGsojaF_15R012453	Wild-specific
SoyGsojaF_15R012761	Wild-specific
SoyGsojaF_15R014785	Wild-specific
SoyGsojaF_15R014784	Wild-specific
SoyGsojaF_15R014867	Wild-specific
SoyGsojaF_15R014866	Wild-specific
SoyGsojaF_15R014879	Wild-specific
SoyGsojaF_18R001211	Wild-specific
SoyGsojaF_18R001311	Wild-specific
SoyGsojaF_18R001309	Wild-specific
SoyGsojaF_18R001310	Wild-specific
SoyGsojaF_18R001308	Wild-specific
SoyGsojaF_18R001763	Wild-specific
SoyGsojaF_18R001762	Wild-specific
SoyGsojaF_18R000502	Wild-specific

SoyGsojaF_18R001931	Wild-specific
SoyGsojaF_19R009438	Wild-specific
SoyGsojaF_19R009439	Wild-specific
SoyGsojaF_19R010672	Wild-specific
SoyGsojaF_02G075400	Wild-specific
SoyGsojaF_02R028181	Wild-specific
SoyGsojaF_20R023985	Wild-specific
SoyGsojaF_20R024168	Wild-specific
SoyGsojaF_20R024227	Wild-specific
SoyGsojaF_04R017078	Wild-specific
SoyGsojaF_04R017079	Wild-specific
SoyGsojaF_06R019710	Wild-specific
SoyGsojaF_06R019774	Wild-specific
SoyGsojaF_06R020173	Wild-specific
SoyGsojaF_06R020860	Wild-specific
SoyGsojaF_08R031731	Wild-specific
SoyGsojaF_08R031790	Wild-specific
SoyGsojaF_08R032197	Wild-specific
SoyGsojaF_08R032522	Wild-specific
SoyZH35_01R003450	Wild-missing
SoyJY_01R005278	Wild-missing
SoyZH13_01G154700.m1	Wild-missing
SoyHF25_10R006394	Wild-missing
SoyHF25_10R006393	Wild-missing
SoyWF7_10R009910	Wild-missing
SoyZH35_10G249000	Wild-missing
SoyZH13_10G263300.m2	Wild-missing
SoyJY_11R053716	Wild-missing
SoyHF25_11R054172	Wild-missing
SoyWF7_11R054047	Wild-missing
SoyZH35_12R050605	Wild-missing
SoyWF7_12R050469	Wild-missing
SoyZH35_13G033900	Wild-missing
SoyWF7_13R041707	Wild-missing
SoyWF7_13R041708	Wild-missing
SoyZH13_14R021171	Wild-missing
SoyZH13_14R021170	Wild-missing
SoyHF25_14R021363	Wild-missing
SoyHX3_14R019661	Wild-missing
SoyHF25_14R023290	Wild-missing
SoyHX3_14R020075	Wild-missing
SoyHX3_14R020076	Wild-missing
SoyHX3_14R020085	Wild-missing
SoyHX3_15G021800	Wild-missing
SoyHF25_15G128200	Wild-missing

SoyWF7_15R007979	Wild-missing
SoyZH13_15G195301.m1	Wild-missing
SoyWF7_15G210300	Wild-missing
SoyHF25_15R017719	Wild-missing
SoyHF25_15R017725	Wild-missing
SoyZH13_15G228201.m5	Wild-missing
SoyZH13_15G228201.m1	Wild-missing
SoyZH13_15G228201.m4	Wild-missing
SoyJY_15R009028	Wild-missing
SoyJY_15G273900	Wild-missing
SoyZH13_15G252001.m1	Wild-missing
SoyHX3_16R055320	Wild-missing
SoyZH35_16R056304	Wild-missing
SoyWF7_16R055704	Wild-missing
SoyWF7_16R055703	Wild-missing
SoyZH35_16G133000	Wild-missing
SoyZH35_16G133500	Wild-missing
SoyZH13_16G121500.m2	Wild-missing
SoyZH35_16R057137	Wild-missing
SoyZH13_16R056845	Wild-missing
SoyJY_16R057848	Wild-missing
SoyHX3_16R056898	Wild-missing
SoyZH35_16R057576	Wild-missing
SoyZH35_16G214800	Wild-missing
SoyHF25_17R051999	Wild-missing
SoyWF7_17R049112	Wild-missing
SoyWF7_17R049111	Wild-missing
SoyJY_17R053016	Wild-missing
SoyHF25_18G067950	Wild-missing
SoyWF7_18R001153	Wild-missing
SoyHX3_18R001641	Wild-missing
SoyZH13_18G142620.m1	Wild-missing
SoyZH13_18G142619.m1	Wild-missing
SoyZH13_18G142622.m1	Wild-missing
SoyZH13_18G142621.m1	Wild-missing
SoyZH13_18G142618.m1	Wild-missing
glyma.Lee.gnm1.ann1.GlymaLee.18G138400.1	Wild-missing
SoyJY_18R002058	Wild-missing
SoyZH35_18R002188	Wild-missing
SoyZH13_18G184700.m1	Wild-missing
SoyHX3_18R002288	Wild-missing
glyma.Lee.gnm1.ann1.GlymaLee.18G201900.1	Wild-missing
SoyZH13_18G226300.m2	Wild-missing
glyma.Lee.gnm1.ann1.GlymaLee.18G216100.1	Wild-missing
SoyZH13_18R002808	Wild-missing

SoyHX3_18R002867	Wild-missing
SoyZH13_18G255801.m5	Wild-missing
SoyZH13_18G256100.m1	Wild-missing
SoyZH13_18G255801.m2	Wild-missing
SoyZH13_18G255801.m1	Wild-missing
SoyWF7_19R026464	Wild-missing
SoyWF7_19R026466	Wild-missing
SoyWF7_19R026465	Wild-missing
SoyHX3_19R024629	Wild-missing
SoyZH35_19R019257	Wild-missing
SoyZH13_19R024841	Wild-missing
SoyHF25_19G134800	Wild-missing
SoyHF25_19R019727	Wild-missing
SoyHF25_19R019726	Wild-missing
SoyHF25_19R019729	Wild-missing
SoyHF25_19R019725	Wild-missing
SoyHF25_19R019728	Wild-missing
glyma.Lee.gnm1.ann1.GlymaLee.19G166300.1	Wild-missing
SoyHX3_02R020791	Wild-missing
SoyZH35_02R023812	Wild-missing
SoyZH35_02R023811	Wild-missing
SoyZH35_02R023813	Wild-missing
SoyZH35_02R023810	Wild-missing
SoyZH35_02R025619	Wild-missing
SoyZH13_02G213501.m1	Wild-missing
SoyZH13_20G069403.m1	Wild-missing
SoyZH35_20R030617	Wild-missing
SoyZH13_20G124203.m1	Wild-missing
SoyZH13_20G124204.m1	Wild-missing
SoyZH13_20G190102.m1	Wild-missing
SoyZH13_20G190900.m1	Wild-missing
SoyHF25_03R029917	Wild-missing
SoyWF7_03G054500	Wild-missing
SoyWF7_03R036143	Wild-missing
SoyWF7_03R036140	Wild-missing
SoyWF7_03R036139	Wild-missing
SoyWF7_03R036148	Wild-missing
SoyWF7_03R036150	Wild-missing
SoyWF7_03R036144	Wild-missing
SoyWF7_03R036151	Wild-missing
SoyWF7_03R036141	Wild-missing
SoyWF7_03R036138	Wild-missing
SoyWF7_03R036142	Wild-missing
SoyWF7_03R036145	Wild-missing
SoyWF7_03R036149	Wild-missing

SoyWF7_03R036137	Wild-missing
SoyWF7_03R036146	Wild-missing
SoyZH13_03G079102.m1	Wild-missing
SoyJY_03R037257	Wild-missing
SoyZH35_03R040535	Wild-missing
SoyZH35_03R040536	Wild-missing
SoyHX3_03R036990	Wild-missing
SoyZH13_03G149100.m1	Wild-missing
SoyJY_03R038167	Wild-missing
SoyZH35_04R013036	Wild-missing
SoyWF7_04R013432	Wild-missing
SoyJY_04R010878	Wild-missing
SoyHF25_04R011263	Wild-missing
SoyZH13_04G168500.m1	Wild-missing
SoyWF7_04R014582	Wild-missing
SoyZH35_05R048920	Wild-missing
SoyHX3_05R046808	Wild-missing
SoyHF25_06R012027	Wild-missing
SoyWF7_06R016682	Wild-missing
SoyZH13_06G219600.m1	Wild-missing
SoyZH13_06G219401.m1	Wild-missing
SoyZH13_06G224704.m1	Wild-missing
glyma.Lee.gnm1.ann1.GlymaLee.06G218000.1	Wild-missing
glyma.Lee.gnm1.ann1.GlymaLee.06G217900.1	Wild-missing
SoyZH35_06R017426	Wild-missing
SoyZH35_06R017425	Wild-missing
SoyZH13_06G239200.m1	Wild-missing
SoyHX3_06R017208	Wild-missing
SoyGsojaF_06R020670	Wild-missing
SoyHF25_06G273500	Wild-missing
SoyZH13_06G253601.m1	Wild-missing
SoyZH13_06G253600.m1	Wild-missing
SoyZH13_06G253700.m1	Wild-missing
SoyHX3_06R017472	Wild-missing
SoyZH13_06G262400.m1	Wild-missing
SoyHX3_07R042134	Wild-missing
SoyHX3_07R042135	Wild-missing
SoyHF25_07R042761	Wild-missing
SoyHF25_07R042762	Wild-missing
SoyHF25_07R042760	Wild-missing
SoyWF7_07R039822	Wild-missing
SoyHX3_07R043911	Wild-missing
SoyHX3_07R043912	Wild-missing
SoyHX3_07R043913	Wild-missing
SoyHX3_07R043914	Wild-missing

SoyZH13_07G202001.m1	Wild-missing
SoyHF25_08R036397	Wild-missing
SoyHF25_08R036396	Wild-missing
SoyWF7_08R033564	Wild-missing
SoyJY_08R035036	Wild-missing
glyma.Lee.gnm1.ann1.GlymaLee.08G248900.1	Wild-missing
glyma.Lee.gnm1.ann1.GlymaLee.08G248800.1	Wild-missing
SoyZH13_08G288600.m1	Wild-missing
SoyZH13_08G288700.m1	Wild-missing
SoyJY_08R035605	Wild-missing
SoyJY_08R035606	Wild-missing
SoyHX3_08R034936	Wild-missing
SoyGsojaF_09R036019	Wild-missing
SoyHX3_09R026643	Wild-missing
SoyHX3_09R026641	Wild-missing
SoyHX3_09R026642	Wild-missing
SoyZH35_09R026895	Wild-missing
SoyHF25_09R027290	Wild-missing
SoyHF25_09R027291	Wild-missing
SoyWF7_09R030703	Wild-missing
SoyHX3_09R027993	Wild-missing
SoyHF25_09G173000	Wild-missing
glyma.Lee.gnm1.ann1.GlymaLee.09G170000.1	Wild-missing
glyma.Lee.gnm1.ann1.GlymaLee.09G170100.1	Wild-missing

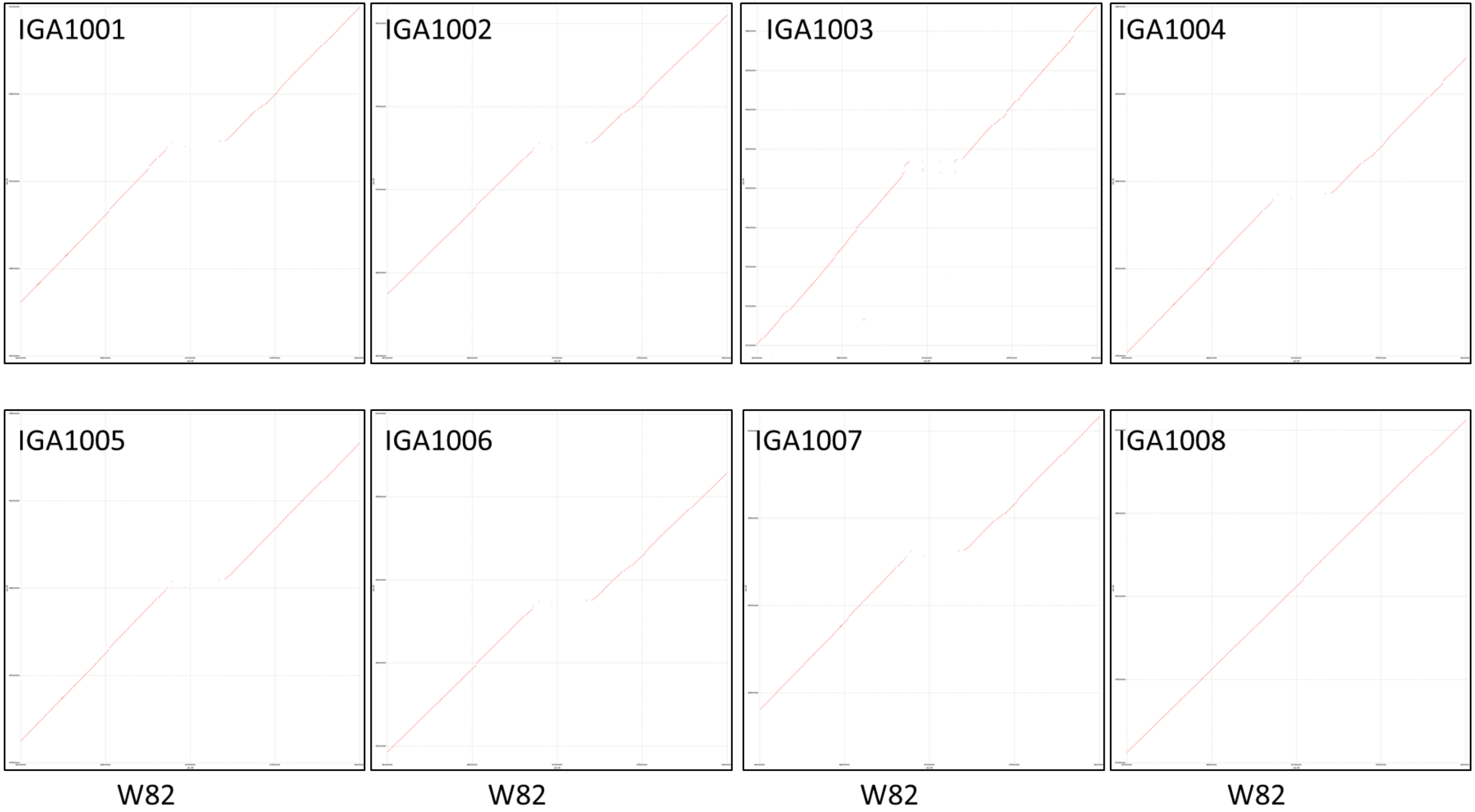

Supplementary Figure 1. Williams 82 specific PAV on chr15 compared with each genome.

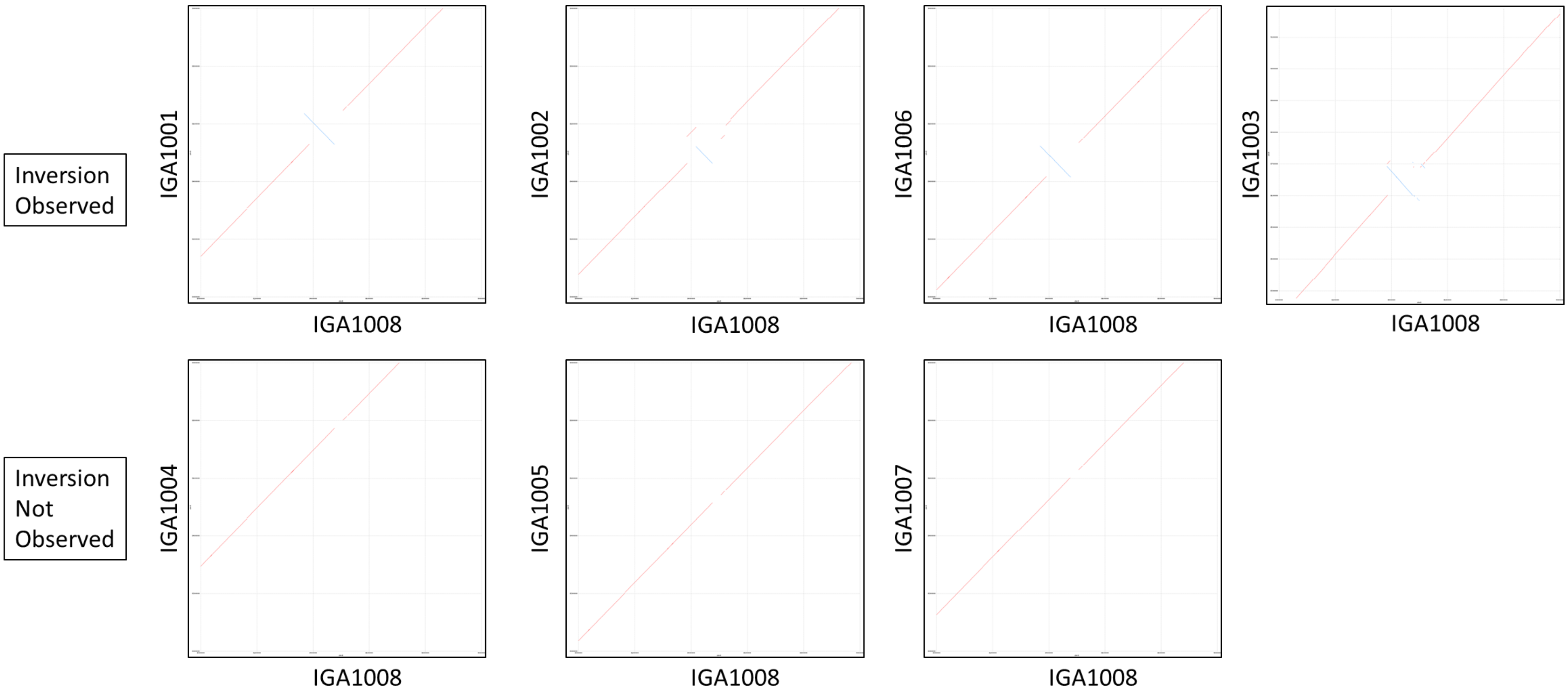

Supplementary Figure 2. Inversion variation on I locus in new genomes compared with IGA1008

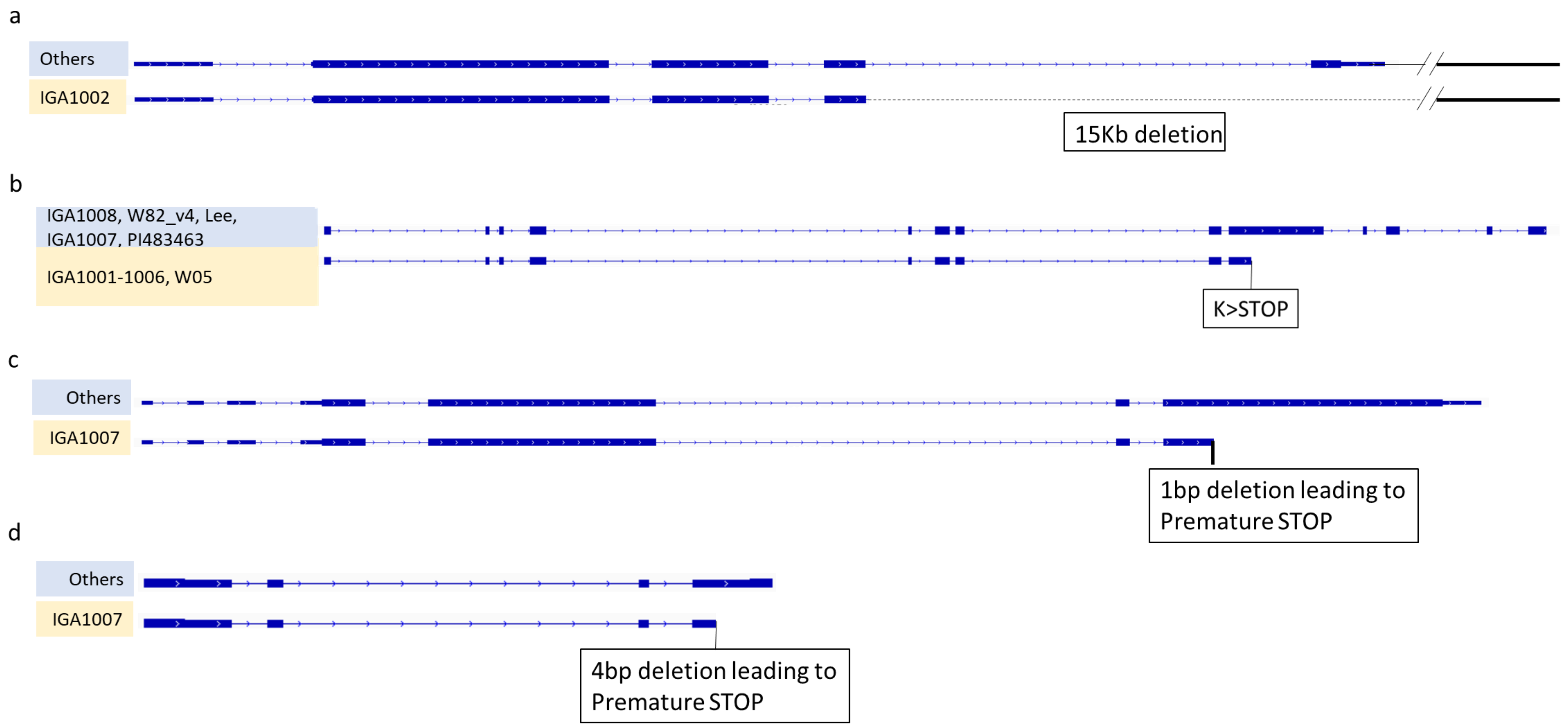

Supplementary Figure 3. Genomic variations found in a) E3 gene, b) E2 gene, c) J gene, d) FT1b gene
